# Phytogenic supplement containing menthol, carvacrol and carvone ameliorates gut microbiota and production performance of commercial layers

**DOI:** 10.1038/s41598-022-14925-0

**Published:** 2022-06-30

**Authors:** Yadav S. Bajagai, Friedrich Petranyi, Sung J. Yu, Edina Lobo, Romeo Batacan, Advait Kayal, Darwin Horyanto, Xipeng Ren, Maria M. Whitton, Dragana Stanley

**Affiliations:** Institute for Future Farming Systems, Central Queensland University, Rockhampton, QLD 4702 Australia

**Keywords:** Bacteria, Pathogens, Microbiology, Molecular biology, Diseases

## Abstract

Consumer push towards open and free-range production systems makes biosecurity on farms challenging, leading to increased disease and animal welfare issues. Phytogenic products are increasingly becoming a viable alternative for the use of antibiotics in livestock production. Here we present a study of the effects of commercial phytogenic supplement containing menthol, carvacrol and carvone on intestinal microbiota of layer hens, microbial functional capacity, and intestinal morphology. A total of 40,000 pullets were randomly assigned to two sides of the experimental shed. Growth performance, mortality, egg production and egg quality parameters were recorded throughout the trial period (18–30 weeks of age). Microbial community was investigated using 16S amplicon sequencing and functional difference using metagenomic sequencing. Phytogen supplemented birds had lower mortality and number of dirty eggs, and their microbial communities showed reduced richness. Although phytogen showed the ability to control the range of poultry pathogens, its action was not restricted to pathogenic taxa, and it involved functional remodelling the intestinal community towards increased cofactor production, heterolactic fermentation and salvage and recycling of metabolites. The phytogen did not alter the antimicrobial resistance profile or the number of antibiotic resistance genes. The study indicates that phytogenic supplementation can mimic the action of antibiotics in altering the gut microbiota and be used as their alternative in industry-scale layer production.

## Introduction

The poultry industry is rapidly growing, fulfilling the global demand for an affordable and healthy protein source. In addition to the rapid growth in advanced economies, the poultry industry is the fastest developing agricultural subsector in developing countries since it can be started with a manageable and affordable investment. The poultry industry is expected to reach $ 422.97 billion in 2025 and increase the growth rate to up to 7% per year^1^. This growth does not come smoothly, and the industry faces several challenges. Large-scale production, animals under high production stress, high pathogen load and heavy exposure to excreta and the environment are bringing animal welfare issues into the spotlight. Additionally, antibiotic use is no longer acceptable because of the surge of antibiotic resistance on farms. Livestock industries had to switch to other methods of controlling the pathogen load in production.

The use of plant-based antimicrobials, also referred to as phytogenics, is one of the most popular pathogen control alternatives. Either alone or in combination with other additives such as probiotics, organic acids, and prebiotics, phytogens ability to control bacterial load is building a respectable reputation in the industry. Supplementation of very diverse types of phytogens, including complex mixes of carvacrol, cinnamaldehyde, capsicum oleoresin, menthol, anethol, eugenol, black cumin seeds, artemisia, oregano, anise, and citrus essential oils, can improve broiler chicken performance^2–6^. In layers, dietary inclusion of phytogens improved a range of critical production parameters such as egg weight^7^ or multiple production indicators including egg weight, egg production, egg mass and feed conversion ratio^8^. Others reported no difference using phytogens^9^.

Across several studies, a range of industry devastating pathogens was reported as reduced by phytogenic supplementation, including *Salmonella*, *E. coli* and *Clostridium*^9^, and many suggested phytogens as comparable to antibiotics in their pathogen control abilities. These effects are also strongly influenced by specific phytogens or combinations used, feed formulation, production system^10^, and are bird gender-specific^11^, affecting male and female birds differently. Other studies show significant interactions of phytogens with sex hormones^12,13^, gut-brain axes^12^, immune parameters^2,3^, enzymes and cholesterol levels^8,14^, to name a few. Phytogenic products can exhibit many other benefits commonly used in human essential oil therapy, such as the calming effect^12^ that can benefit the poultry industry, where pecking and aggression contribute significantly to overall mortality. On the other hand, phytogens are known to show side effects such as cytotoxicity^15,16^, especially in higher doses or prolonged use.

Although in many countries, livestock industries, including broiler production, can still use low-level antibiotics in the feed if needed, the layer industry is somewhat specific in that it cannot consider antibiotics addition into the feed because of the possible antibiotic residue in the egg. Once the birds must be antibiotic-treated for the disease, the eggs often cannot go into human consumption. This makes the egg industry more dependent on phytogenic and other alternative pathogen control. Many products on the market show different levels of pathogen control, influence on the productivity parameters, and various additional benefits and side effects of common phytogens. In this study, we performed an industry scale experiment investigating the effects of a commonly used phytogenic supplement on a range of the production, functional, and intestinal health parameters.

## Results

### Animal health and performance

**Figure 1:**
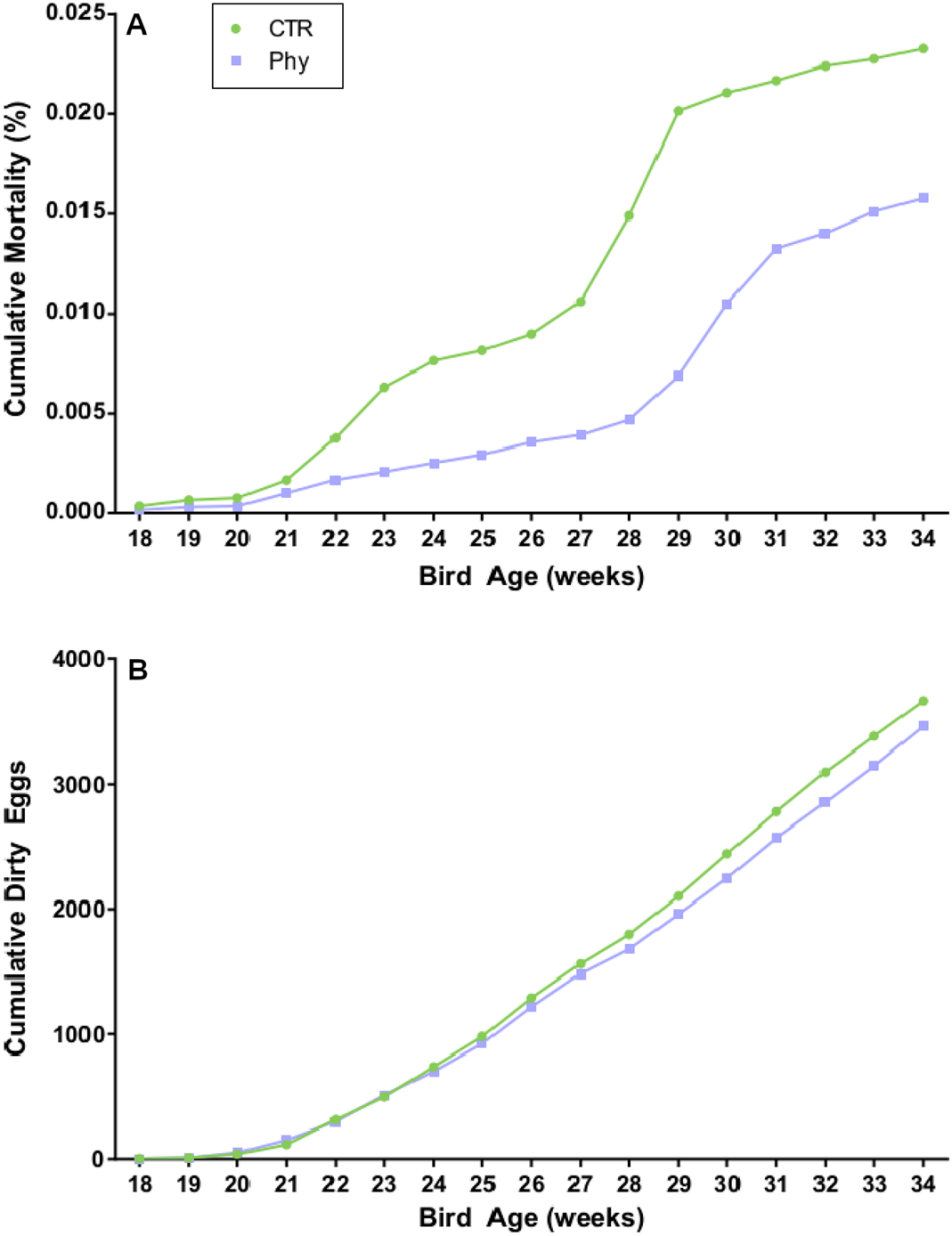
Cumulative mortality and a cumulative number of dirty eggs show a continual trend of improvement in the phytogen supplemented group.

The layer flocks on both sides of the shed exhibited good performance within the standards expected on the farm for the time of the year and the breed. The weekly measurements of the parameters described in the methods section showed an overlap in most measured categories; however, there was a clear and persistent trend of lower mortality and dirty eggs in the phytogen supplemented group (Fig. 1). The dirty eggs parameter was measured to monitor the levels of diarrhoea and runny excreta as an indirect indicator of gut health instead of gut scoring. The enteric gut scoring is the option preferably evaded because it requires killing the bird and is considered when poor gut health is apparent and diagnostic tests are needed. Other performance parameters measured are provided in Fig. S1 of the supplementary material.

Histological analysis showed no significant difference in villi morphology and short chain fatty acid (SCFA) analysis showed no significant differences in concentrations of acetic, propionic, butyric, isobutyric, valeric and isovaleric acid using Mann–Whitney test and P < 0.05 cutoff.

### Phytogen supplementation and pathogen control

**Figure 2:**
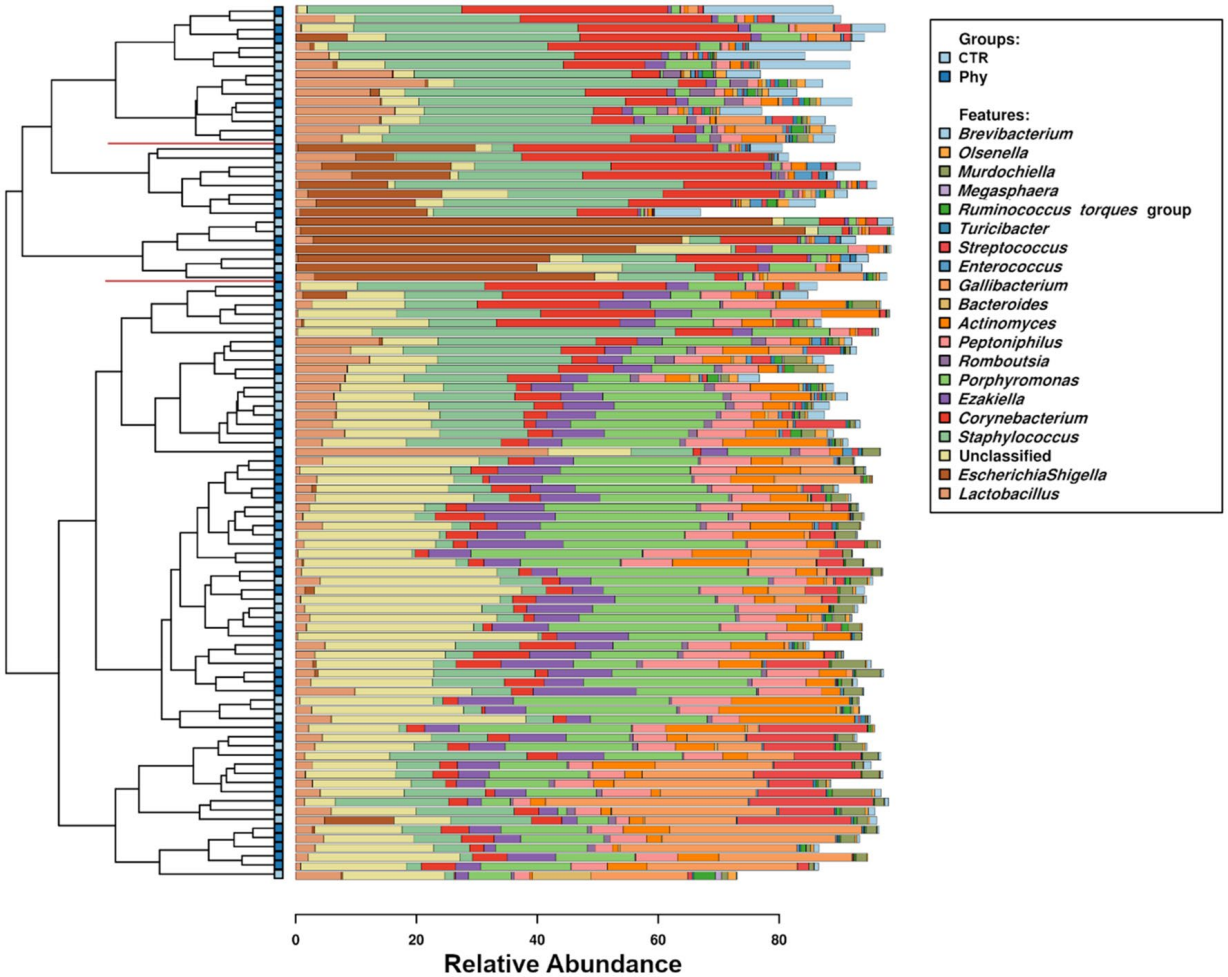
Top 20 most abundant genera and their distribution in cloacal swab microbiota. Red lines on the row dendrogram indicate *Escherichia* dominated cluster of birds.

The microbial community, estimated by 16S rRNA analysis of cloacal swabs, was strongly affected by the phytogenic supplementation assessed by RDA multivariate statistics, with P < 0.001. The most abundant genera included beneficial *Lactobacillus*, *Ruminococcus*, *Bacteroides*, *Faecalibaterium* and *Butyricicoccus*, undesirable *Escherichia*, *Staphylococcus*, *Gallibacterium* and others shown in Fig. 2. Red lines in Fig. 2 point at the cluster of birds highly dominated by *EscherichiaShigella* genus (SILVA taxonomy). Core microbiota analysis identified 11 genera unique to the control shed, including *Virgibacillus*, *Pseudoscardovia*, *Peptococcus*, *Megasphaera*, *Lachnoclostridium*, *Glutamicibacter*, *Fusobacterium, Erysipelotrichaceae* UCG002*, Erysipelatoclostridium*, *Enorma* and *Aeriscardovia*. The core analysis was done to estimate shared microbiota, with a presence in at least 40% of birds in the group as a cutoff value.

**Figure 3:**
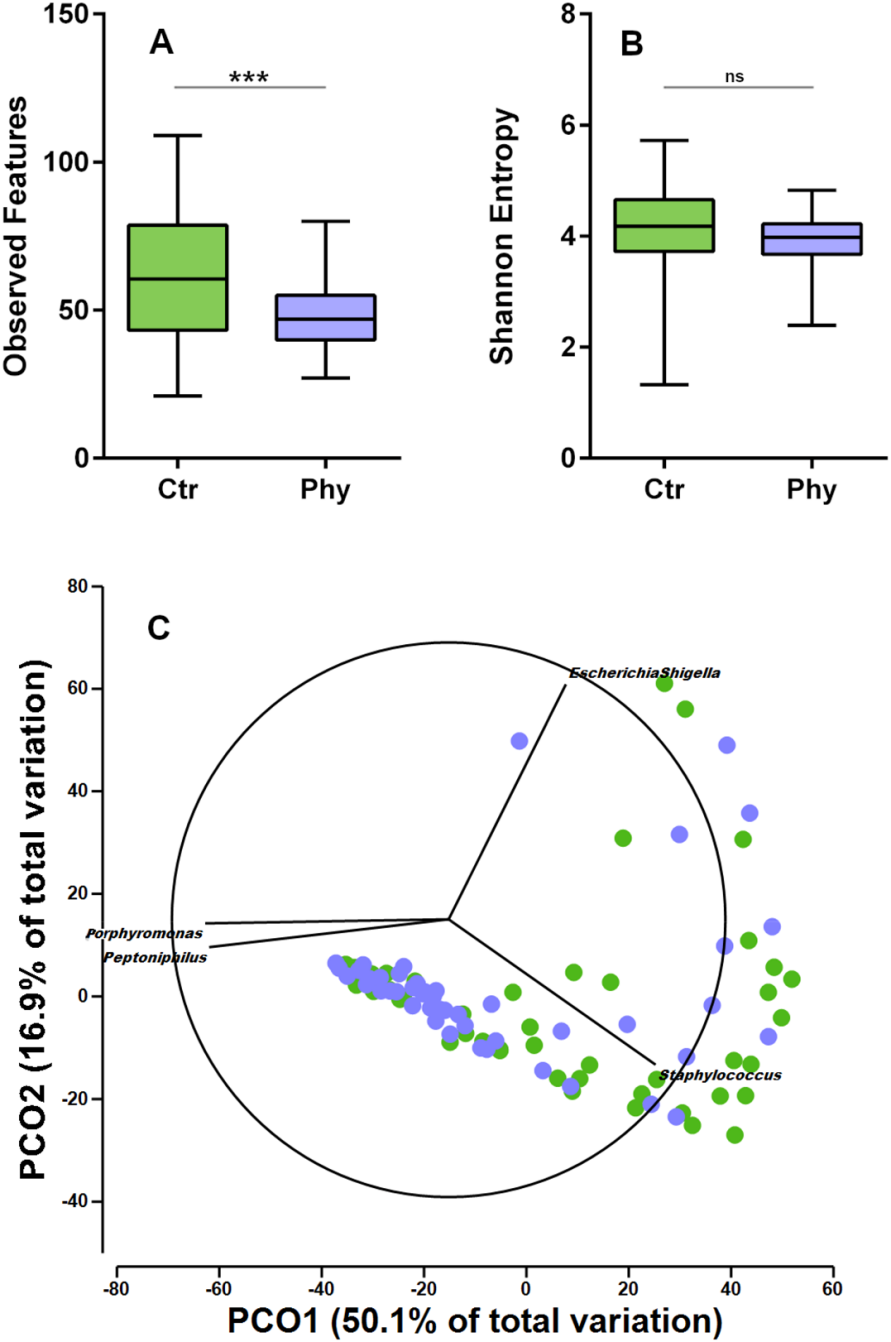
Alpha and beta diversity measures. The number of observed features was significantly reduced in phytogen supplemented birds. PCoA plot is generated in Primer 7-e using genus-level data and Bray Curtis distance. The plot displays genus-level data with a Pearson correlation higher than 0.8 genera shown as vectors.

Alpha diversity indicators point to a significant reduction (Mann–Whitney test *P* = 5.09e^−4^) of the Observed Features index in the phytogen group, with the trend of reduced richness extending to a genus level with *P* = 3.7e^−4^, while other parameters, including Evenness, Shannon and Simpson, were not significantly altered (Fig. 3).

Beta diversity analysis also indicates significant alterations of microbial structure introduced by phytogen supplementation with Adonis statistics suggesting significant differences between the two communities (by Weighted Unifrac *P* = 0.048 and Bray Curtis *P* = 0.001). PCoA plot presented in Fig. 3 uses Bray Curtis to compare the sample-to-sample distance at the genus level and displays higher grouping in phytogen supplemented samples and dispersion of control microbial structures.

### Genera affected by phytogen

**Figure 4:**
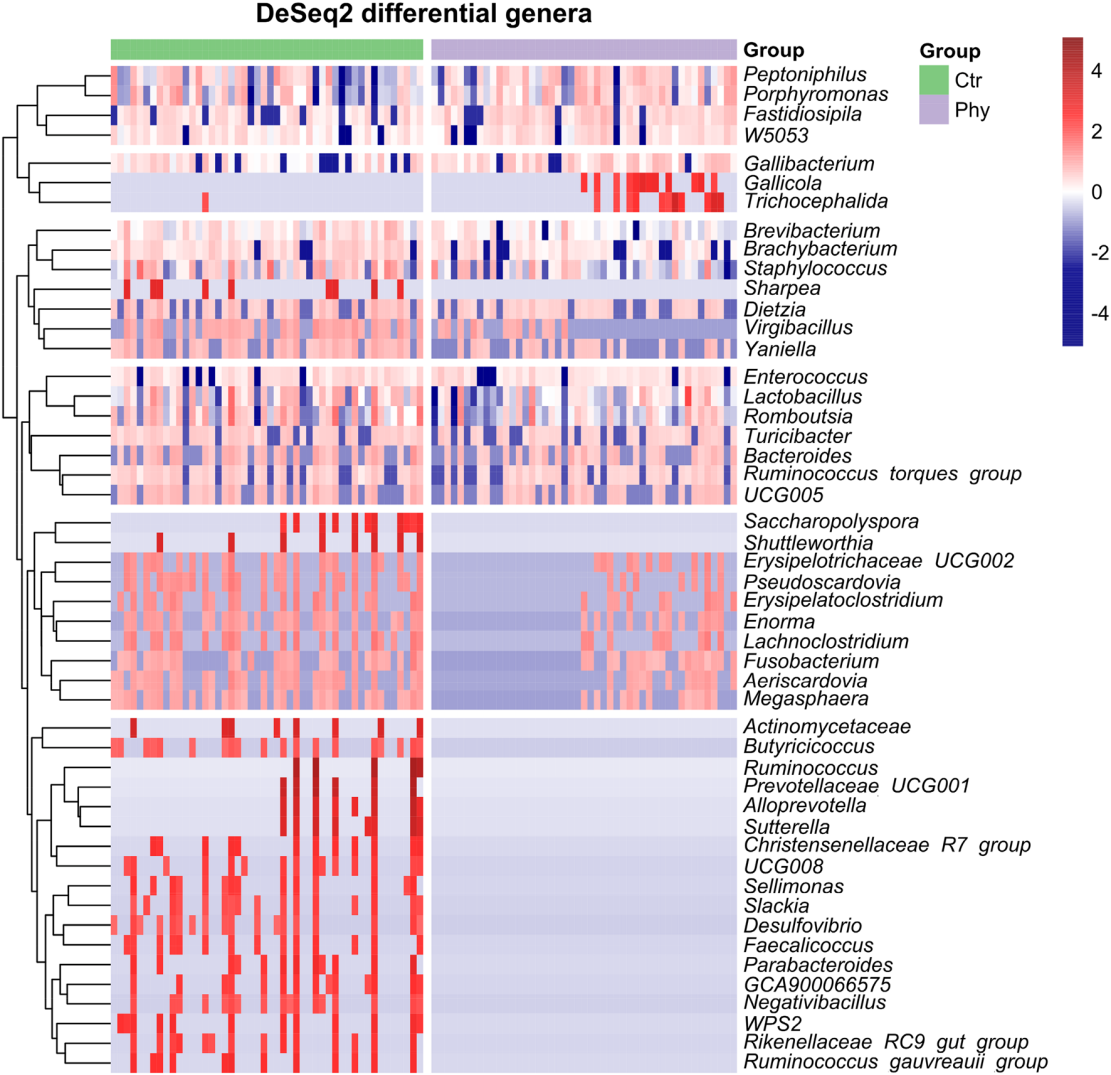
DeSeq2 selected significant genera. The top 2 clusters represented by seven genera are increased in abundance in the phytogen supplemented group. The heatmap was generated in the pheatmap R package using mean scaled, log2 transformed abundance data with the normalised range from low (blue) to high (red) abundance shown on the scale bar.

Out of 49 swab genera identified as significantly altered (DeSeq2, *P* < 0.05, Fig. 4), only seven were increased by phytogen supplementation (*Gallicola*, *Trichocephalida*, *Fastidiosipila*, *Gallibacterium*, *Peptoniphilus*, *W5053* and *Porphyromonas*) represented as the top two clusters on the heatmap (Fig. 4). Remaining genera, including *Butyricicoccus, Negativibacillus, Desulfovibrio, Faecalicoccus, Slackia, Sellimonas, Virgibacillus, Aeriscardovia, Rikenellaceae RC9 gut group, Saccharopolyspora, Parabacteroides, Ruminococcus gauvreauii* group, *Christensenellaceae R7* group*, Dietzia, Actinomycetaceae, Lachnoclostridium, Yaniella, Enterococcus, Megasphaera, Pseudoscardovia, Romboutsia, Sutterella, Bacteroides, Alloprevotella, Sharpea, Fusobacterium, Shuttleworthia, Enorma, Staphylococcus, Turicibacter, Lactobacillus, Erysipelatoclostridium, Brachybacterium, Ruminococcus, Brevibacterium* and others shown in Fig. 4 heatmap were reduced in the phytogen supplemented group.

**Figure 5:**
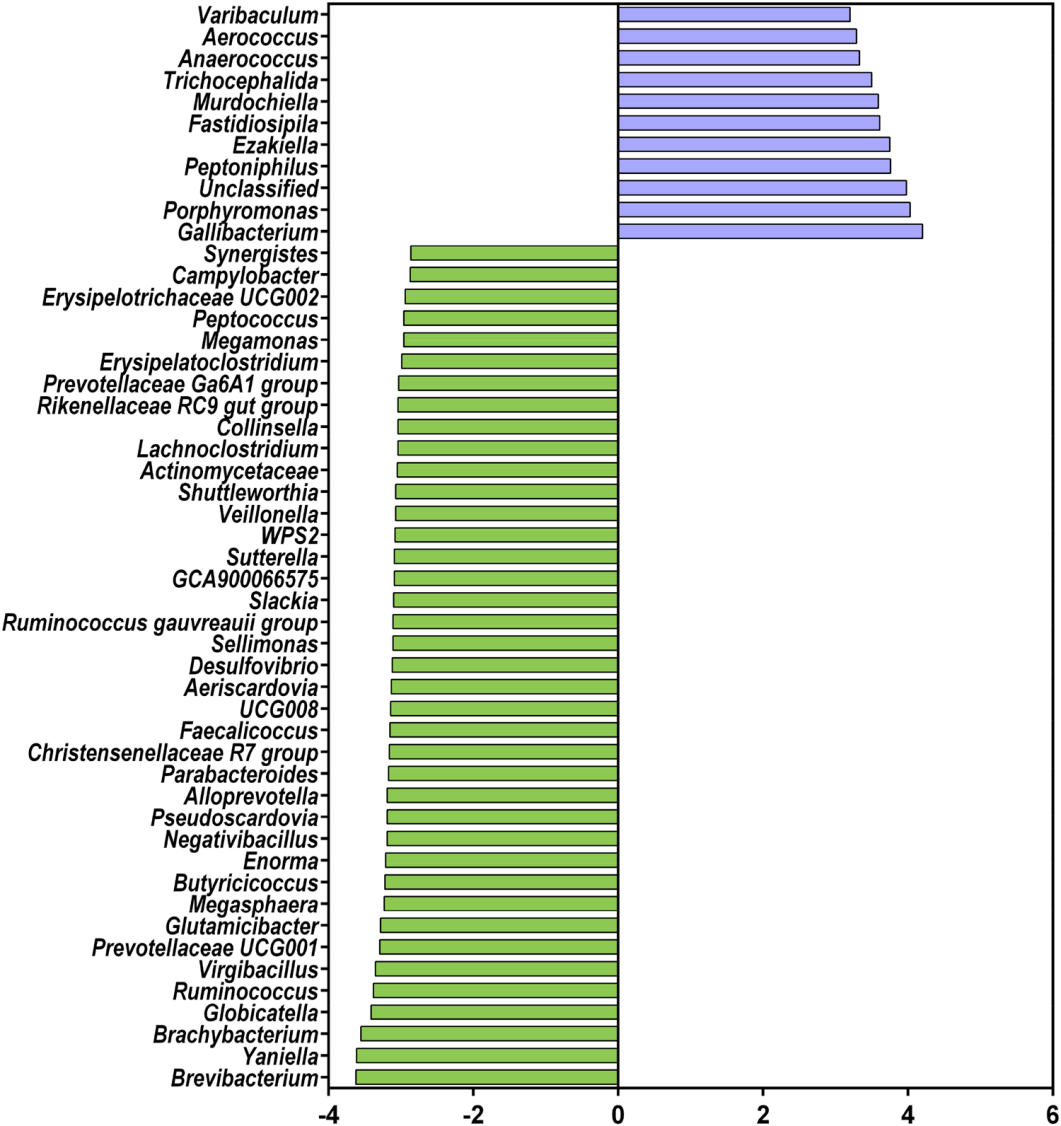
LefSe plot of genus level (SILVA taxonomy) differential taxa.

Similarly, the linear discriminant analysis (LDA) effect size (LEfSe) selects comparable genera cohorts as biomarker level representatives of the microbial communities of the two groups, adding welfare-significant *Campylobacter* as a control group marker genus (Fig. 5).

### Functional capability and phytogen supplementation

**Figure 6:**
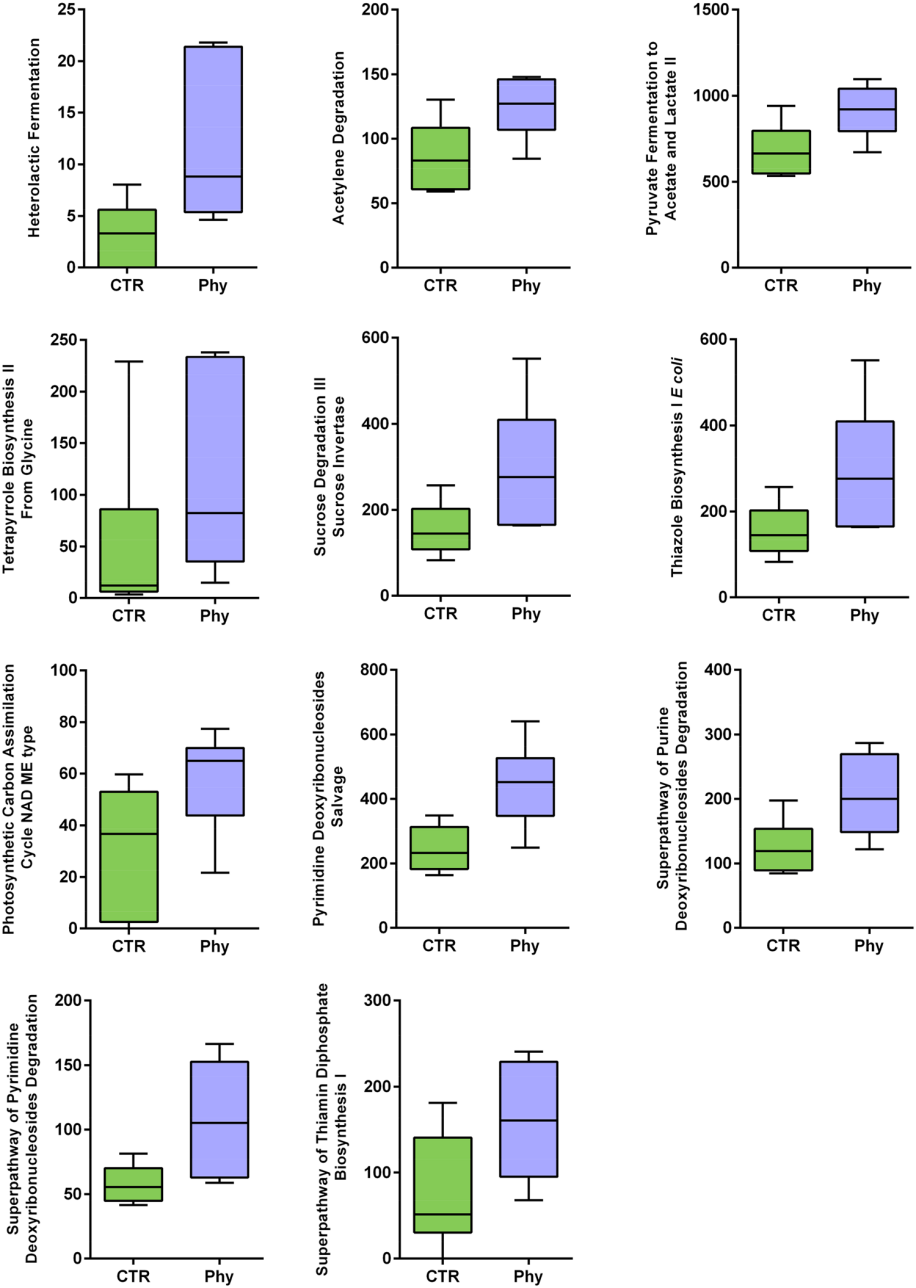
Differentially abundant functions (*P* = 0.0087–0.041).

Metagenomic sequencing showed slight differences in the functional profiles that were insignificant between the two groups, with RDA *P* = 0.344 (Fig. 6). Wilcoxon ranked tests selected 11 individual functions as significantly affected by phytogen supplementation: pyrimidine deoxyribonucleosides salvage, heterolactic fermentation, sucrose degradation III sucrose invertase, superpathway of thiamin diphosphate biosynthesis I, photosynthetic carbon assimilation cycle NAD ME type, thiazole biosynthesis I *E. coli*, tetrapyrrole biosynthesis II from glycine, pyruvate fermentation to acetate and lactate II, superpathway of pyrimidine deoxyribonucleosides degradation, superpathway of purine deoxyribonucleosides degradation, acetylene degradation, as shown in Fig. 6.

### Resistome analysis

**Figure 7:**
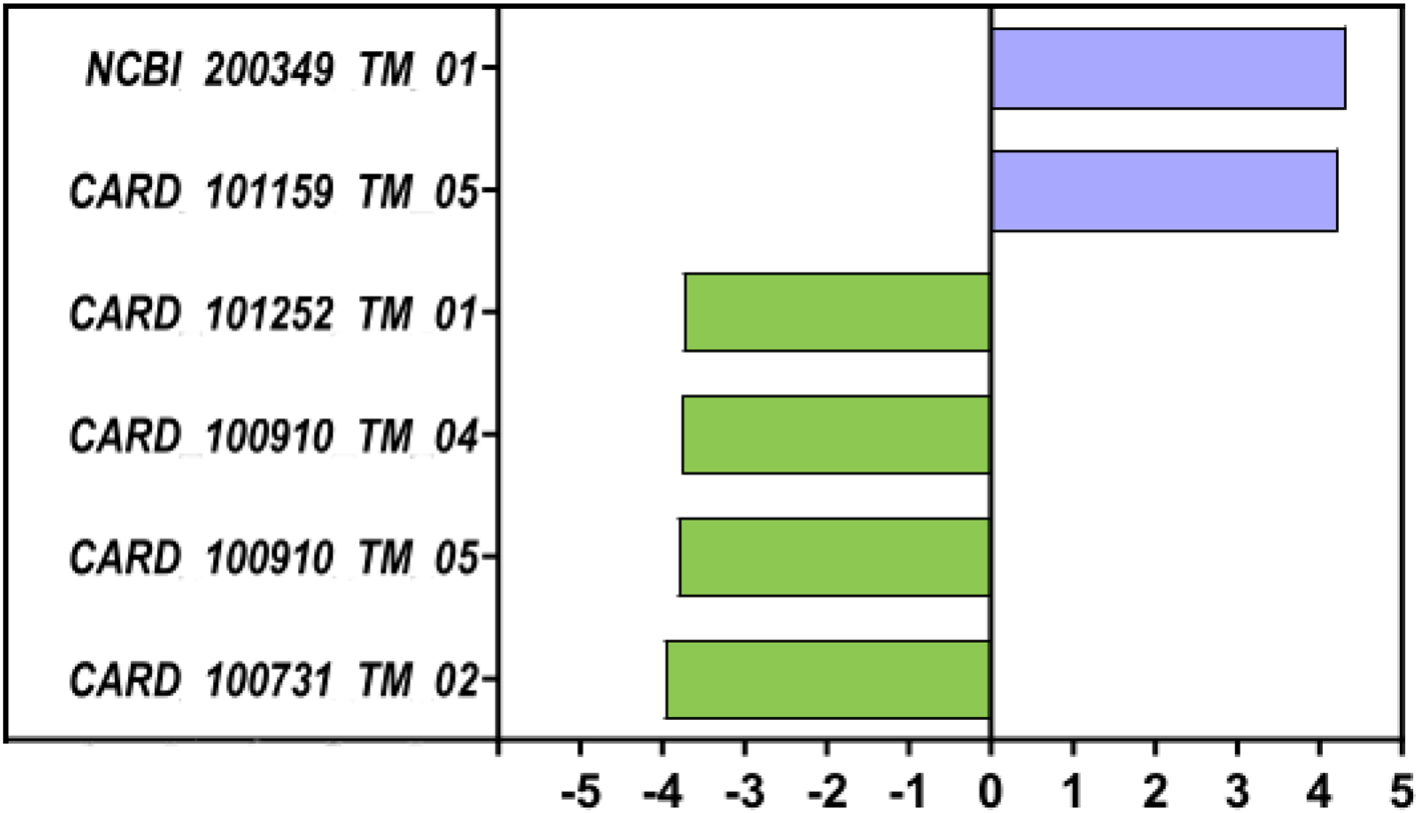
LefSe analysis of the resistome profile indicated 6 AMR conferring genes as altered by phytogen, two associated with phytogen (purple) and four with control birds (green).

Resistome analysis against QMI-AR database showed no significant difference in resistome profiles RDA *P* = 0.282. There were no significant differences in the total number of antimicrobial resistance (AMR) genes (AMR richness as an observed number of genes *P* = 0.77). LefSe biomarker discovery analysis shown in Fig. 7, indicated alterations in 6 AMR genes, 4 of them are tetracycline resistance-related genes, one gene (NCBI 200349 TM 01) conferring resistance to erythromycin, telithromycin, and streptogramin was associated with phytogen and another gene (CARD 101252 TM 01) conferencing resistance to ribostamycin, butirosin, kanamycin A, gentamicin B, paromomycin, lividomycin A, lividomycin B, plazomicin, neomycin, G418 and aminoglycoside antibiotics was associated with control birds.

## Discussion

Although the present study represents a single industry-based experiment, the trends and findings are highly obstinate and relevant for the egg industry. The trend of reduced mortality and a lower number of dirty eggs that persisted for 16 weeks could be related to pathogen control and improvements in intestinal health. Unlike other studies that reported significant improvements in egg size or quality^7–9^ we recorded variable and overlapping egg parameters (supplementary data S1). More experiments are needed to confirm the reproducibility of mortality and dirty eggs improvement as these parameters have a high significance in animal welfare.

The alpha and beta diversity measures and the so-called "core" analysis to identify highly shared genera indicating loss of ASVs (isolate level taxa) and genera in the phytogen supplemented group with a significant drop in richness at both ASV and genus level. This is in agreement with phytogen supplemented birds showing more grouping in ordination plots indicating reproducible microbiota remodelling via phytogenic supplementation and general ability to reduce the number of different taxa resemble antibiotic effects^17^. Similarly to antibiotics, there is also no selective removal of just pathogenic taxa; like in antibiotics, some beneficial gut commensals are expected as collateral damage.

Our study used regularly updated Silva database taxonomy, and SILVA taxonomic assignments are majorly different to the Green Genes picked taxonomy^18^ used in the majority of the previous literature. For example, the *Clostridium* genus has been reclassified and split into multiple genera to be able to separate beneficial from pathogenic clostridia, and the taxonomy outputs using the two databases can differ to a highly incomparable level, as investigated by others^18^ and observed in our analysis. SILVA, however, is up to date with the rapid development of culturomics and novel species and genera emerging, and some well-known taxa being reclassified. The abundance of newly emerged genera and species discovered via advancing culturomics makes the data interpretation more exciting and more challenging.

In this experiment, the birds had access to the outdoor environment, and consequently, the pathogen load was challenging. Interestingly while other potentially pathogenic genera were evenly distributed across the birds on both control and phytogen, some pathogens like *Escherichia* and *Corynebacterium* formed clusters of birds dominated by these pathogens. The bar chart in Fig. 2 singles out the 2 dendrogram subclusters enriched with *EscherichiaShigella* genus (brown) with the bottom subcluster almost fully dominated by *EscherichiaShigella*, and the upper subcluster of birds is showing *EscherichiaShigella* co-domination with *Corynebacterium* (red). Similarly, there was a group of birds dominated by *Staphylococcus*. These birds make likely candidates for flock mortality.

The phytogen increased only seven genera and suppressed 42 (Fig. 1). *Porphyromonas* was one of the induced genera and was also among the 20 most abundant (Fig. 2). Not much is known about the interactions of this genus and chicken. One of the members of this species *P. gingivalis* is a Gram-negative anaerobic bacterium common in oral microbiota that can overcolonise the subgingival region resulting in a bad, putrid mouth odour. A number of studies used egg yolk immunoglobulin (IgY) produced in egg yolks after chickens were challenged^19^ or immunised^20^ with *P. gingivalis* that could control and prevent oral diseases caused by *P. gingivalis* as an effective treatment for halitosis. There are no benefits reported from any of the members of this genus; its many species are members of human or animal oral microbiota associated with oral pathogenesis or emerging pathogens like *Porphyromonas levii* commonly reported in cattle rumen and several veterinary clinical cases, including bovine interdigital necrobacillosis^21^.

*Gallibacterium* was another pathogenic genus increased in the phytogen supplemented group. It is known by the *Gallibacterium anatis*, a common cause of reproductive tract infections, reduced egg production and increased mortality and highly prevalent and fast speeding in layer flocks^22^. It was suggested that *Gallibacterium* is a good indicator of management and biosecurity, however, with more access to open range consumer push, biosecurity becomes impossible to control as birds have access to wild birds, insects, rodents and soil, all major sources of pathogens. *Gallicola* is a common chicken gut genus recently isolated^23^ with no indications of pathogenicity. *Fastidiosipila* was a genus described in 2005^24^ and reported as common in chicken manure^25^. ASVs assigned to *Fastidiosipila* by SILVA, are among those assigned to *Clostridium* genus in Green Genes database. Another relatively recently annotated genus *Peptoniphilus*^23^ are common genital microbiota in humans^26^ and also very recently reported in swine feces^27^.

Among the genera reported as suppressed by phytogen supplementation, we have a few generally accepted beneficial genera such as *Lactobacillus*, *Ruminococcus* and *Butyricicoccus* and a wide range of genera with known and emerging pathogenic behaviours. While we had only two poultry pathogens increased in the phytogen supplemented group, the list of pathogens removed or reduced in the supplemented group is impressive. The most clinically significant in poultry production is *Campylobacter*, identified by LefSe as a marker for control birds.

Other pathogens reduced in the treatment group include pathogenic *Fusobacterium*^28^, *Desulfovibrio*^29,30^ and *Slackia*^31,32^. *Saccharopolyspora* brings a new dimension to pathogen control as this genus is the principal cause of Farmer's Lung Disease (FLD)^33^. *Actinomyces* sp. cause actinomycosis, a chronic, granulomatous infectious disease^34^. In a recent study, intratumor bacteria of the *Lachnoclostridium* genus ranked top in a positive association with infiltrating CD8 + T cells^35^, was reduced in colorectal cancer (CRC)^36^, and in another study it was increased in IBS^37^. *Enterococcus* is also a pathogenic genus with *Enterococcus cecorum* as poultry commensal bacteria and opportunistic pathogen causing outbreaks of enterococcal spondylitis ("kinky back") in poultry, with strains pathogenic in a range of other host animals. It frequently causes co-infection with *E. coli* in chicken, resulting in avian colibacillosis^38^. In *Enterococcus faecium* some strains are pathogenic and others beneficial^39^. *Alloprevotella*, an oral microbiota member, has recently been linked with opportunistic infection^40^. There is numerous literature on pathogenic effects on the heart, brain and liver^41–43^ and cancer-promoting effects of *Fusobacterium*^44^ and its role in colitis^45^, gut pathogenesis and abundance of virulence factors^46^. *Erysipelatoclostridium* has one species known as highly pathogenic with a range of clinical situations, including bacteraemia, septicaemia, brain and lung abscess and other infections spanning across the host organs and tissues. It was previously known as *Bacillus ramosum* and *Clostridium ramosum* and renamed in 2013 to *Erysipelatoclostridium ramosum*^47,48^. Another pathogenic genus reduced by phytogen is *Brachybacterium*; this genus was first reported as a cause of bloodstream infection in 2018^49^, with subsequent pathogenicity reports continuing^50,51^ to emerge. Similarly, *Brevibacterium* is involved in opportunistic and systemic infections^52–55^. *Staphylococcus* is a universally known pathogen reduced by phytogen supplementation. *Dietzia* is also pathogenic^56,57^ and has broad applications in biotechnology.

There were a number of more recently named genera, including *Yaniella* genus proposed in 2008^58^, *Pseudoscardovia* species were also more recently isolated from wild pigs in 2013 and 2014, members of *Bifidobacteriaceae* family but are also not highly investigated species^59,60^. Reclassification of *Romboutsia* genus from *Clostridium* was proposed in 2014^61^. *Shuttleworthia* became a genus in 2002^62^, *Enorma* in 2013^63^, *Sutterella* species started to emerge recently with a recent report that it may be IGA degrading species in ulcerative colitis^64^. Similarly, there is not much in the literature on the *Sharpea*, *Erysipelotrichaceae UCG002*, *Prevotellaceae UCG001*, *Ruminococcus torques* group. *Megasphaera* are common in humans and agricultural animal microbiota, while in chicken ceca*, Megasphaera statnotii* was recently isolated butyrate producer^65^.

Phytogen supplementation increased functional capability across 11 functional categories. Deoxyribonucleotides are synthesised de novo, and this process is very costly for the cell resources and energy, so many organisms, including bacteria and plants, have the ability to salvage ribonucleotides and reduce energy wastage. This process is often limited to the salvage of individual components of the pathways, and it has a critical role in cell division, especially in G1 stage^66^ (Metacyc database). An increase in superpathway of pyrimidine deoxyribonucleosides degradation and superpathway of purine deoxyribonucleosides degradation are overlapping functions with intersecting outcomes.

The heterolactic fermentation pathway refers to the lactic bacteria that are normally divided into homofermentative that produce lactate and heterofermentative that additionally produce a range of other metabolites. The phytogen supplementation influenced this balance tilting it towards heterofermentative pathway enrichment, which could be expected to result in increased production of other metabolites, including ethanol, acetate, glycerol and carbon dioxide^67^. Pyruvate fermentation to acetate and lactate II is another pathway that would lead to acetate and lactate, either amplifying the effects or resulting in overlapping enzymes involvement. However, acetic acid was not significantly changed in cecal microbiota.

Sucrose degradation III sucrose invertase pathway, also increased in phytogen supplementation, is a pathway resulting in the production of hexoses that feed into starch biosynthesis and glycolysis IV pathways. Additionally, increased biosynthesis of thiamine vitamin B1, is essential for energy metabolism. B1 is an essential cofactor for a diversity of critical enzymes. Thiazole biosynthesis I *E. coli* is also B1 related pathway. An increase in tetrapyrrole biosynthesis II from glycine can be another link to vitamin B and enzyme functions. Tetrapyrroles function as metal-binding cofactors in many vital enzymes, including heme and cobalamine (vitamin B12).

Despite showing the ability to keep bacterial populations from overgrowth by reducing richness and the ability to control a range of pathogens, the phytogenic product did not significantly alter AMR profile nor increase the richness (number) of AMR genes in the supplemented community. The alterations of a very few AMR genes can be easily attributed to microbiota change as AMR genes are "stored" within bacterial species, and every additive that alters microbiota will have to alter AMR profile by reducing AMR genes contained in species it reduces and increasing the abundance of AMR genes in the species it promotes. However, these phytogenic products generally reduced the pathogen abundance and removed the bacterial richness, thus more likely to reduce the total abundance of AMR genes in the community, which cannot be quantified with the current methodology.

## Conclusion

Combined with the shift in microbiota, functional analysis points at the remodelling of taxa with overlapping functional effects that would result in the changed metabolic profile of lactic acid bacteria products, improved cell energy and resource conservation, and likely enhanced thiamine and enzyme cofactor availability. Reduction of a range of pathogens and overall bacteria-suppressing ability makes phytogens one of the most promising alternatives to antibiotics.

## Materials and methods

### Ethics statement

The Animal Ethics Committee of the Central Queensland University approved the study under the approval number 0000020312. All methods were carried out according to the Australian Code for the Care and Use of Animals for Scientific Purposes. All methods used in the animal trial are reported in accordance with Animal Research: Reporting of In Vivo Experiments (ARRIVE) guidelines and regulations.

### Experimental design

The experiment was performed in one of Australia's leading egg-producing farms, and one of the specially designed research sheds was used to test the productivity of various products offered to the layer industry. The shed was physically split into two independent sections (control and treatment), each housing 20,000 birds (total 40,000) and each section on the same batch of feed, with the phytogenic product being the only difference (shed diagram is provided in Fig. S2 of the supplementary material). The wall physically separated the two shed sections, and the mesh wire separated the two sides of the shed in the open range. The birds arrived from the raring shed at 18 weeks of age and came from the same batch of pullets. Birds were randomly assigned to control or phytogenic treatment. The feed used was designed by the company nutritionist to meet the production and animal welfare requirements, and it was mainly composed of sorghum and lower levels of other grains such as wheat and barley. The protein sources used in the diet were soybean meal and a lower inclusion of meat and bone meal. A standard premix composition was also added to the diet in order to cover the mineral and vitamin bird requirements.

The commercial phytogenic supplement used in the study (Digestarom®, DSM) contained a mix of essential oil extracts and herbs with menthol, carvacrol and carvone as major bioactive compounds. The supplement was added to the feed at the concentration of 150 g/t phytogenic product.

Performance measures routinely collected by the company on a weekly basis include mortality (daily and cumulative dead birds), rate of lay (ROL, percentage measured from the daily amount of eggs produced divided by the number of birds in the shed), cumulative hen housed eggs (amount of eggs laid divided by the initial amount of birds placed at a point of lay), grams of feed consumed per bird per day (GBD, measured using weight cells under the sheds silos retrieving the total feed used per day and per side of the shed), feed conversion (kg of feed used to produce one dozen eggs), bird body weight (weighing randomly 100 birds per side of the shed), cumulative dirty eggs, egg weight, eggshell thickness, eggshell Haugh units, average yolk colour (evaluated at 1–16 according to the Differential Colorimeter yolk colour chart**)**. Haugh unit, yolk index, egg weight, eggshell strength, yolk colour and eggshell thickness were measured using Digital egg tester DET6000 (NABEL Co., Ltd, Japan).

When the birds were 30 weeks of age and reached the peak of egg-laying production, cloacal swabs were taken from 50 birds on each control and treatment for microbiota analysis. Ten random birds were selected from each group, and intestinal sections were separated and selected for other types of analysis. Cecal contents were collected from 10 birds for metagenomic analysis and SCFA analysis, and a mid-ileum intestinal section for histology.

### Histology

The tissue samples of the ileum midsection were collected and fixed in 10% buffered formalin solution. Fixation, paraffin embedding, deparaffinisation and rehydration were outsourced to a commercial medical pathology company (PathCare, Australia). The staining was performed with hematoxylin and eosin (H&E). For each tissue sample, 20 well-oriented villi and crypts were selected for morphometric measures. The measured parameters were: villus height (distance from the tip to the bottom of the villi), villus width (mean value between basal and apical villi width) and the crypt depth (distance between the crypt neck and its base).

Statistical analysis of the results obtained in the experiment was carried out using statistical software GraphPad Prism version 9.3.0 (GraphPad, San Diego, CA, USA).

### Short-chain fatty acids

The standards and cecal samples were analysed on a GC–MS (GC–MS–QP2010 Ultra) fitted with a AOC-20 s Shimadzu autosampler and a Shimadzu AOC-20i auto-injector with a polar column (Agilent J&W GC). SCFAs were determined by injecting a 1 µl sample with helium as the carrier gas. The mass spectrometer operated in the electron ionisation mode at 0.2 kV, the source temperature was 220 °C.

### DNA extraction

DNA was extracted as previously described^10,68^. Briefly, swabs were lysed using the method adapted for 16S microbiota analysis described by Yu and Morrison^69^, and purified using a DNA spin purification column (Enzymax LLC, Cat# EZC101, Kentucky, US). The DNA quality and quantity were estimated using a NanoDrop spectrophotometer.

### Sequencing and data analysis

Primers used for amplification of the V3-V4 region of 16S rRNA genes were: forward ACTCCTACGGGAGGCAGCAG, reverse GGACTACHVGGGTWTCTAAT. The primers contained barcodes, spacers and Illumina sequencing linkers^70^. The sequencing library preparation was performed following the manufacturer's protocol (Illumina Inc., San Diego, CA, USA). Sequencing was completed on the Illumina MiSeq platform using 2 × 300 bp paired-end sequencing.

The data was analysed using Quantitative Insights Into Microbial Ecology 2 (QIIME 2)^71^. Demultiplexing was performed using Cutadapt^72^. Trimmed QIIME 2 demux file had Phred quality scores of minimum 15, 25th Phred 37, median Phred 37, 75th Phred 37 and maximum Phred 39. Dada2^73^ was used to error correct, and chimera cleaned the data and the input sequence file was truncated to a uniform length of 200 nt with other Dada2 parameters kept as default. Taxonomy was assigned using the SILVA database^74,75^. The resulting ASV table was trimmed to the minimum sample size of 3697 and a maximum of 26,407 sequences per sample. Five samples were discarded from the analysis due to the small sample sequence size leaving 95 swab samples presented in the data analysis. There was no statistical difference between the groups in the number of sequences per sample. Data analysis and interpretation were made using Hellinger transformed^76^ ASV table at an ASV and genus level.

Primer-e v7 and Calypso v8.72^77^ were used to further explore and present the data. Performance data was plotted and analysed using GraphPad Prism and R packages pheatmap, DeSeq2, vegan and phyloseq were also used in the data exploration.

Metagenomic sequencing was performed on Illumina NovaSeq platform with 2 × 150 bp paired-end configuration sequencing six control and six phytogen cecal DNA samples to a total number of 423 million sequences at a minimum depth of 27 million and a maximum of 43 million quality-filtered sequences. Sequences contained zero ambiguous bases, minimum phred score of 22 and sequences smaller than 150nt were discarded. The taxonomic profiling was done with MetaPhlan2, which uses clade-specific marker sequences bundled in ChocoPhlAn, functionally annotated species pangenomes. HUMAnN2 and UniRef (universal protein reference database) were used. HUMAnN2 used Bowtie 2 for nucleotide level pangenome mapping and Diamond for translated search in unmapped reads.

Resistome analysis was done using CLC Genomic Workbench 21, Microbial Genomics module and ShortBRED algorithm against The QIAGEN Microbial Insight Antimicrobial Resistance (QMI-AR) database. QMI-AR database contains peptide markers derived from the major AMR databases: CARD (https://card.mcmaster.ca/), ARG-ANNOT (http://backup.mediterranee-infection.com/article.php?laref=282&titre=arg-annot), NCBI Bacterial Antimicrobial Resistance Reference Gene Database, (https://www.ncbi.nlm.nih.gov/bioproject/PRJNA313047) and ResFinder (https://bitbucket.org/genomicepidemiology/resfinder_db/src/master/).

## Supplementary Information

Supplementary Figures.

## Data Availability

The amplicon and metagenome sequence data are publicly available at The National Center for Biotechnology Information (NCBI) Sequence Read Archive (SRA) database (https://www.ncbi.nlm.nih.gov/sra) under the BioProject Accession Number PRJNA834577.
