# Supplementary material for: Phytogenic supplement containing menthol, carvacrol and carvone ameliorates gut microbiota and production performance of commercial layers
Source: Sci Rep. 2022 Jun 30;12:11033. doi: 10.1038/s41598-022-14925-0 (PMC9246849; doi:10.1038/s41598-022-14925-0)
Supplement: Supplementary file 1 — Supplementary Figures. [file 41598_2022_14925_MOESM1_ESM.pdf]

Phytogenic supplement containing menthol, carvacrol and carvone ameliorates gut microbiota and production performance of commercial layers

Yadav S. Bajagai[1], Friedrich Petranyi[1], Sung Yu[1], Edina Lobo[1], Romeo Batacan Jr[1], Advait Kayal[1], Darwin Horyanto[1], Xipeng Ren[1], Maria De Las Malvinas Whitton[1], Dragana Stanley[1,*]

[1]Central Queensland University, Institute for Future Farming Systems, Rockhampton, Queensland, 4702, Australia

Supplementary File 1

[1]Central Queensland University, Institute for Future Farming Systems, Rockhampton, Queensland, 4702, Australia

[§]Corresponding author
Dragana Stanley
Central Queensland University
Institute for Future Farming Systems
Rockhampton QLD 4702, Australia
Telephone: +61 7 4923 2079
E-mail: D.Stanley@cqu.edu.au

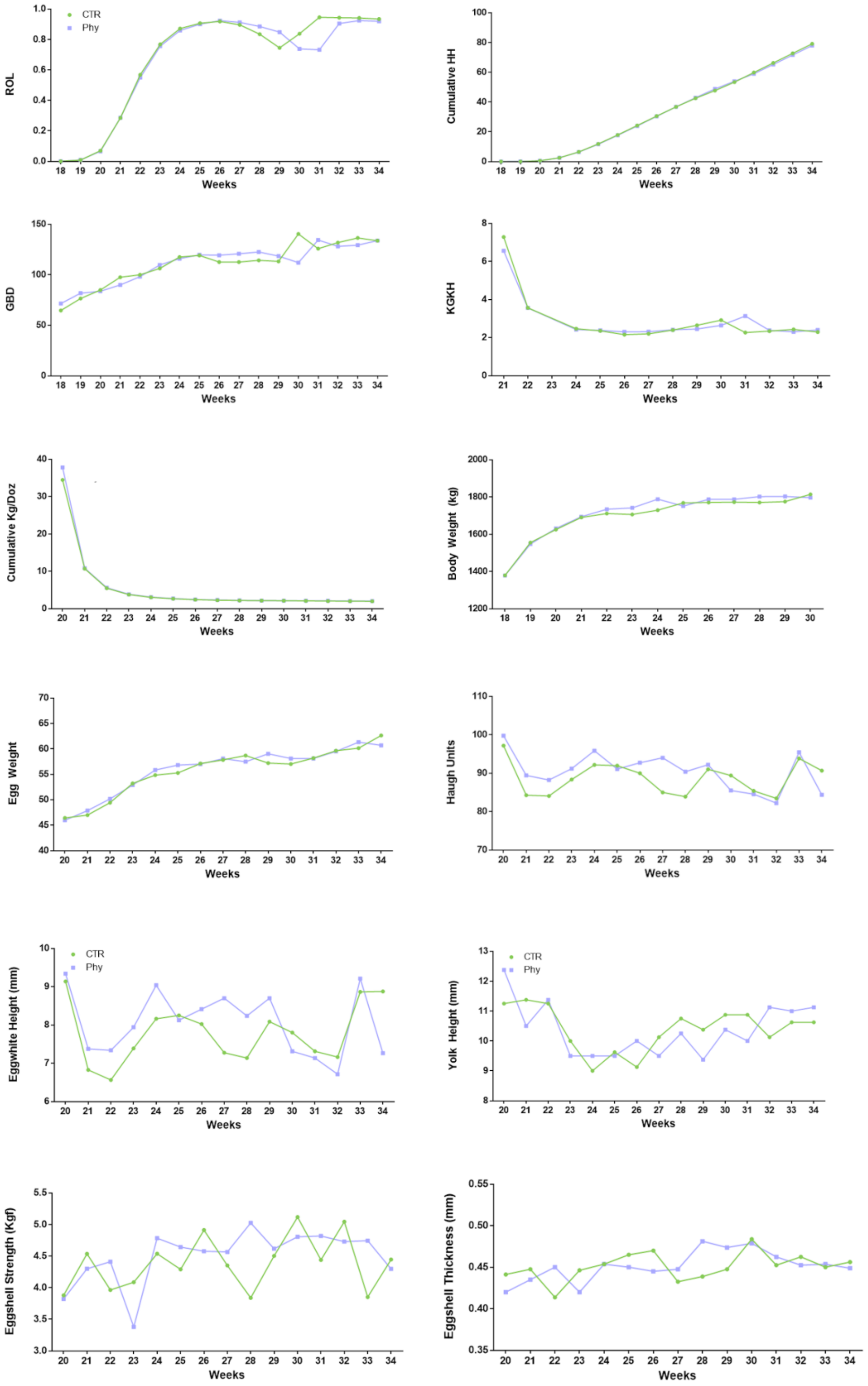

Supplementary Figure 1: Performance measures: Rate of Lay (ROL); Cumulative Hens Housed (HH); grams of feed consumed per bird per day (GBD); KGKH; feed conversion given as cumulative kg of feed used to produce one dozen eggs = kg/doz), bird body weight, egg weight, Haugh units, eggwhite height, yolk height, eggshell strength and eggshell thickness’

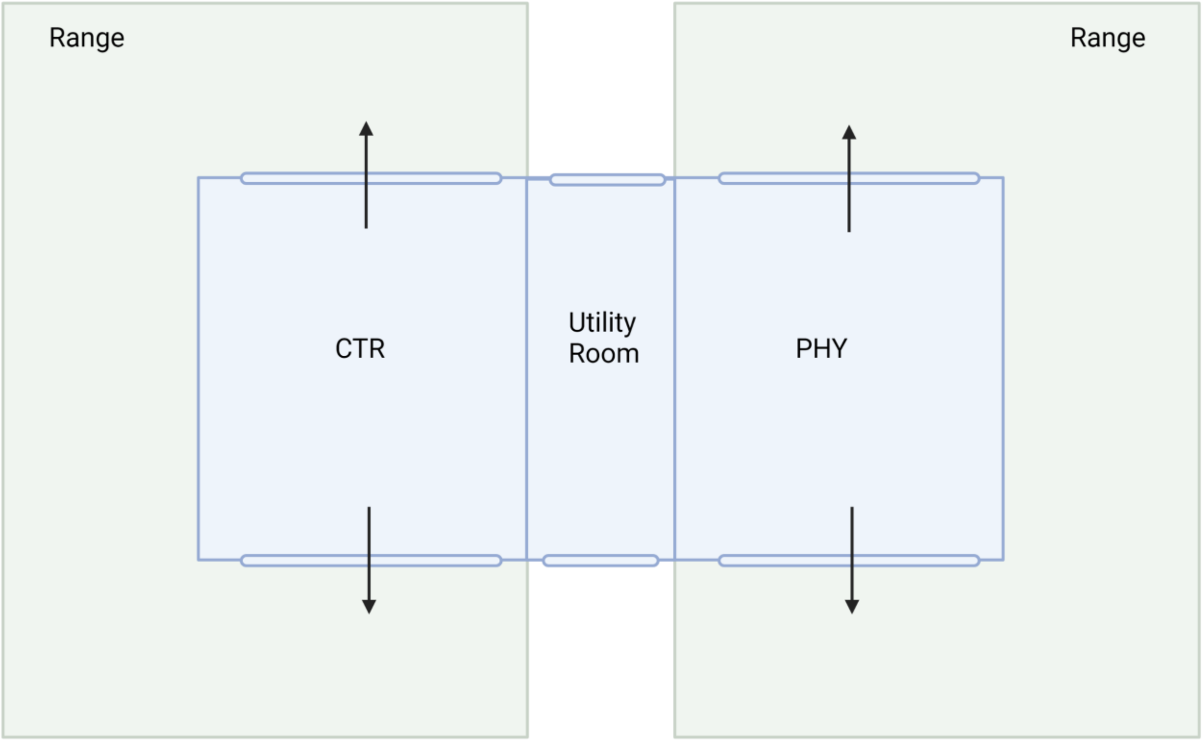

Supplementary Figure 2: Shed design: Blue colour indicates the shed and green open range. Arrows point to exit roller doors that allow the range access. The birds had no contact in the shed or outside.
